# A Duty-Cycled PLL and Fractal Antenna Co-Design Architecture for a Low-Power IR-UWB Transmitter in Neural Implants

**DOI:** 10.3390/s26134241

**Published:** 2026-07-04

**Authors:** Wenjun Zou, Jie Yang, Mohamad Sawan

**Affiliations:** 1 Department of Physics, Fudan University, Shanghai 200433, China; zouwenjun@westlake.edu.cn; 2 Center of Excellence in Biomedical Research on Advances-on-Chips Neurotechnologies (CenBRAIN Neurotech), School of Engineering, Westlake University, Hangzhou 310030, China

**Keywords:** biosensors, neural implants, wireless transmitter, IR-UWB, low power, PLL, compact antenna

## Abstract

We present in this paper a low-power impulse-radio ultra-wideband (IR-UWB) transmitter architecture for neural implants. It features a duty-cycled phase-locked loop (PLL) and a co-designed compact fractal antenna. To suppress the carrier frequency drift inherent in open-loop ring oscillators while maintaining ultra-low power consumption, a hybrid PLL-oscillator upconversion scheme integrated with a switch-controlled voltage-holding module is proposed. Operating at a 10% duty cycle, the PLL consumes merely 90 μW and achieves a locking frequency of 4.25 GHz with a peak-to-peak jitter of 2.14 ps. Furthermore, to eliminate the bulky output matching network, an 8 mm × 10 mm coplanar-waveguide-fed fractal antenna is co-designed to present the conjugate impedance required by the power amplifier output, significantly advancing the miniaturization and energy efficiency of the neural implant. The complete transmitter was fabricated in TSMC 40 nm CMOS, with a supply voltage of 1.0 V, and in vitro wireless experiments through 18 mm of porcine tissue validated the design with a total power consumption of 0.58 mW.

## 1. Introduction

Neural implants have emerged as revolutionary technologies for decoding neural activities and restoring impaired sensory or motor functions in patients with severe neurological disorders. As the demand for high-resolution brain mapping drives the number of simultaneous recording channels from hundreds to thousands, the massive volume of acquired neural data imposes a severe bottleneck on the wireless telemetry module. Traditional narrowband wireless transmission technologies, such as Bluetooth Low Energy (BLE), Zigbee, and the Medical Implant Communication Service (MICS), suffer from limited data rates and degraded energy efficiency, making them inadequate for high-density neural recording. In contrast, impulse-radio ultra-wideband (IR-UWB) technology, characterized by its inherently wide bandwidth, high data rate, ultra-low power consumption, and minimal tissue thermal damage, has emerged as a highly promising technique for next-generation implantable wireless communications [1,2,3,4,5,6,7,8,9,10,11].

Despite these advantages, realizing highly reliable IR-UWB transmitters within the stringent power and area constraints of neural implants remains a formidable task. A primary obstacle is the stability of the carrier frequency. Conventional ultra-low-power IR-UWB transmitters typically employ open-loop voltage-controlled ring oscillators (VCROs) to generate the carrier signal. However, such open-loop topologies are highly susceptible to process, voltage, and temperature variations, resulting in substantial long-term frequency drift that risks violating the Federal Communications Commission (FCC) spectrum emission regulations [12,13].

To address this frequency instability, integrating a PLL is an intuitive solution. Analog PLLs are characterized by superior phase noise performance and the absence of quantization noise compared to digital PLLs. For low-power wireless transmission in implants, the analog implementation avoids the high-frequency sampling clock required by complex digital loop filters and ensures a cleaner transmitter (TX) output spectrum [14,15]. However, due to output oscillation for the mixer, conventional PLLs used for upconversion often need to remain continuously active and consume mW-level power [16,17,18,19]. This leads to significantly higher power consumption compared to switchable oscillators that can enter a sleep mode when not transmitting data, thereby limiting the operational lifespan of the neural implant [20,21].

Another critical limitation arises from the excessive silicon area occupied by the output matching network (OMN). In standard radio-frequency transmitter designs, the power amplifier (PA) must be impedance-matched to a conventional 50 Ω antenna load. Within the UWB spectrum, achieving this 50 Ω match necessitates bulky on-chip inductors and capacitors. These passive components typically consume more silicon area than the entire active transmitter core, thereby severely restricting the miniaturization of the implant.

**Figure 1:**
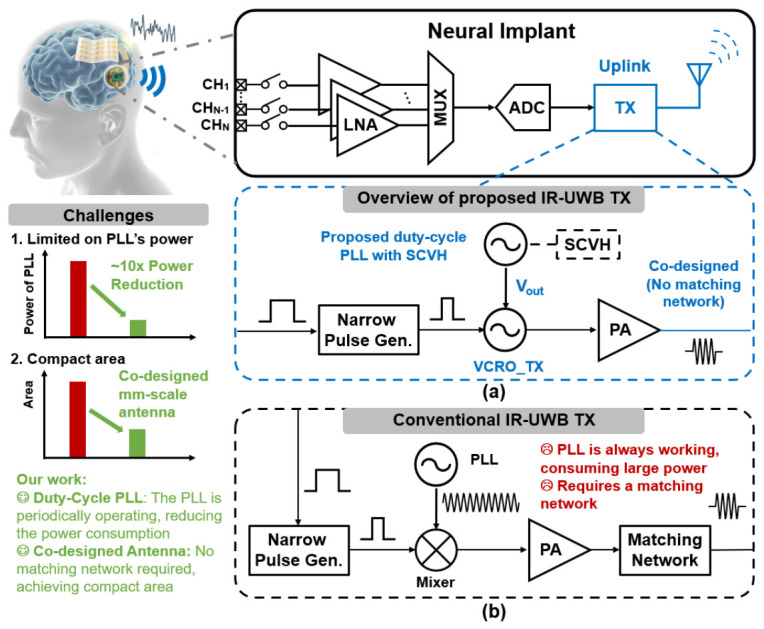
(**a**) Block diagram of a neural implant including an IR-UWB wireless transmitter, and proposed IR-UWB-TX scheme utilizing a duty-cycled PLL with SCVH and a co-designed mm-scale antenna. (**b**) Conventional IR-UWB TX scheme requiring continuous PLL operation and a matching network.

To overcome these design bottlenecks, we propose a novel low-power duty-cycled PLL and fractal antenna co-design architecture in Figure 1. By exploiting the slow-varying nature of the in vivo environment, a switch-controlled voltage-holding (SCVH) module is introduced, enabling the PLL to operate in a low-duty-cycle calibration mode. This operation reduces power consumption by 89% while seamlessly maintaining a stable 4.25 GHz carrier. Crucially, rather than relying on an area-consuming OMN to match a 50 Ω load, a system-level co-design methodology is adopted. A highly miniaturized, 8 mm × 10 mm coplanar waveguide (CPW)-fed 4th-order fractal antenna is specifically engineered to directly present the conjugate impedance required by the PA. This holistic approach completely eliminates the OMN, drastically reducing the implant’s volume.

The remaining parts of this paper provide a comprehensive overview of the proposed PLL architecture and circuit implementation in Section 2, the antenna co-design methodology in Section 3, the post-layout simulation and measurement results in Section 4, followed by a summary in Section 5.

## 2. Proposed Low-Power PLL Design

### 2.1. Low-Power PLL Architecture

**Figure 2:**
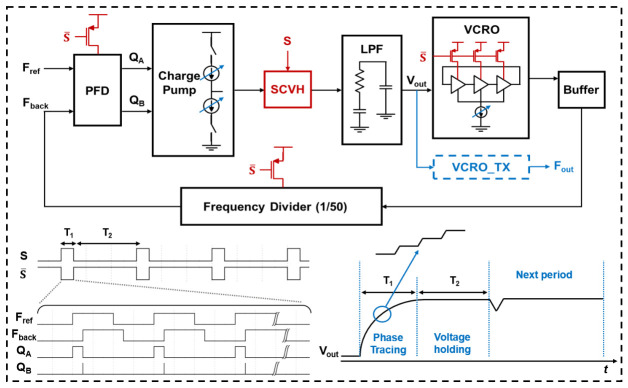
Architecture and timing diagram of the proposed PLL.

The proposed low-power PLL architecture, illustrated in Figure 2, employs a novel dual-oscillator scheme to achieve duty-cycled operation. The system comprises a phase-frequency detector (PFD), a charge pump (CP), a low-pass filter (LPF), a frequency divider, a SCVH module, and two identical VCROs. One VCRO operates within the PLL’s feedback loop, while the second (VCRO_TX) is dedicated to the transmitter’s upconversion stage.

The PFD detects whether the reference signal $F_{ref}$ leads or lags the feedback signal $F_{back}$, generating corresponding phase-difference outputs $Q_{A}$ and $Q_{B}$. These outputs drive the CP and LPF to produce a control voltage $V_{out}$ that adjusts both VCRO frequencies identically. The controlled voltage $V_{out}$ is simultaneously fed to the identical VCRO_TX for the upconversion of the transmitter.

The key innovation of the proposed architecture is its duty-cycled calibration-silent operation mode enabled by the SCVH. In the calibration phase $T_{1}$, the SCVH remains active, enabling normal PLL operation and voltage updates. The PFD compares $F_{ref}$ and $F_{back}$, and the CP and LPF jointly generate the control voltage $V_{out}$, ensuring frequency locking of both VCROs. In the silent phase $T_{2}$, the SCVH is switched off to isolate and hold $V_{out}$. Meanwhile, the PFD, frequency divider, and in-loop VCRO cease operation, and the CP is consequently turned off, thereby achieving near-zero power consumption during $T_{2}$. Notably, when the PFD is deactivated, both phase-difference outputs $Q_{A}$ and $Q_{B}$ settle to zero, which synchronously turns off the switching transistors $M_{1}$ and $M_{2}$ in the CP. This further suppresses charge leakage attributable to the $C_{ds}$ and $C_{gs}$ of transistors $M_{5}$ and $M_{6}$.

For correct functionality, $T_{1}$ must exceed the PLL’s locking time to guarantee phase convergence, while $T_{2}$ depends on the drift tolerance of $V_{out}$ and ambient temperature variation. This time-multiplexed PLL design is particularly well-suited for systems such as neural implants, where power budgets are highly constrained and signal transmission occurs intermittently.

### 2.2. Circuit Implementation

#### 2.2.1. Switch-Controlled Voltage-Holding Module

**Figure 3:**
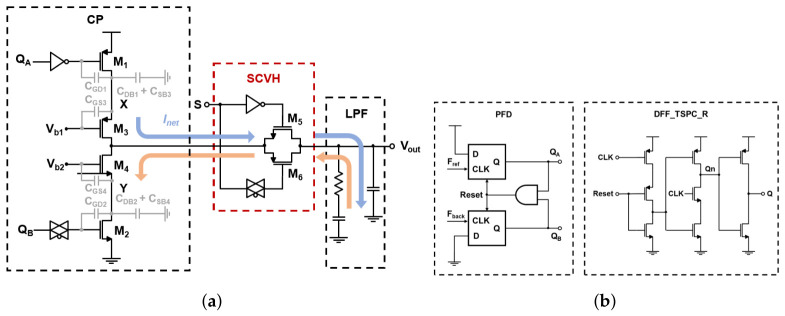
Circuit implementation of (**a**) the proposed CP, SCVH, and LPF, and (**b**) PFD and DFF_TSPC_R.

The phase-difference signals $Q_{A}$ and $Q_{B}$ generated by the PFD are fed into the charge pump to produce the charge/discharge current $I_{net}$, which in turn regulates the control voltage $V_{out}$. However, due to the gate-drain overlap capacitance of the switching transistors, the rising and falling pulses from $Q_{A}$ and $Q_{B}$ will couple through the gate-drain capacitance, introducing disturbances to $V_{out}$. To mitigate this clock feedthrough effect, the switching transistors $M_{1}$ and $M_{2}$ in Figure 3a are placed at the supply and ground terminals, respectively. Therefore, the total capacitance from nodes X and Y to ground helps suppress the coupling effect before any change occurs at the source nodes of $M_{3}$ and $M_{4}$ (which act as current sources).

The SCVH module is implemented using a transmission gate composed of PMOS and NMOS transistors, controlled by the signal S. When S=1, the transmission gate is turned on, allowing the current $I_{net}$ to flow into or out of the LPF. When S=0, the SCVH is turned off, isolating the charge pump from the LPF and enabling voltage holding on $V_{out}$. To suppress skew caused by the misalignment of the pull-up and pull-down pulses, a transmission gate is inserted at the gate of $M_{2}$ and $M_{6}$ to introduce a delay equal to that of an inverter, ensuring synchronized pulse arrival.

#### 2.2.2. PFD

The PFD employs two True Single-Phase Clock (TSPC) D flip-flops (DFFs) and a reset logic to compare the phase and frequency difference between the reference clock $F_{ref}$ and feedback clock $F_{back}$. As shown in Figure 3b, DFF_TSPC_R is a modified version of the TSPC DFF that incorporates a reset function to support rapid clearing, a key requirement for reliable PFD operation.

The TSPC DFF utilizes a single-phase clocking scheme, eliminating the reliance on complementary clock signals typically required by conventional master–slave DFFs. This helps reduce the impact of clock skew on performance. Additionally, the TSPC DFF is well-suited for high-frequency and low-power applications, aligning with our design goals.

#### 2.2.3. Divider

**Figure 4:**
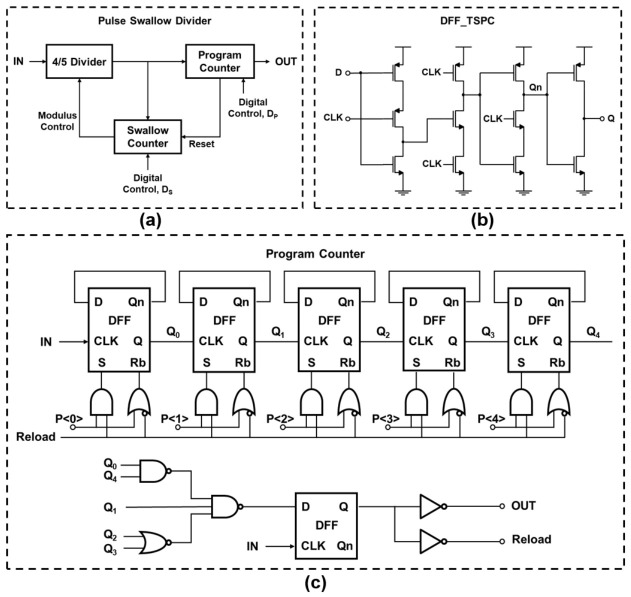
Circuit implementation of Pulse Swallow Divider, DFF_TSPC, and Program Counter.

The frequency divider, with its division ratio set to 50, is realized by a Pulse Swallow Divider, as shown in Figure 4. This flexible integer-N frequency divider achieves programmable division ratios by combining a dual-modulus prescaler (divide-by-4/5), a swallow counter, and a program counter. The divider begins in the higher division mode (divide-by-5) and remains in this state for 2 cycles defined by the swallow counter. Once the swallow counter resets, the prescaler switches to the lower division mode (divide-by-4) and continues until the program counter completes its count. The total division ratio is determined by 4P+2, where *P* is the program counter value.

## 3. Transmitter and Antenna Co-Design

### 3.1. Elimination of On-Chip Matching Networks

A critical challenge for implantable IR-UWB transmitters is the antenna design, where size, bandwidth, gain, and integration must be simultaneously optimized. Conventional on-chip matching networks occupy significant silicon area and introduce insertion losses. The co-design approach eliminates the area-intensive on-chip matching network by designing an antenna whose input impedance is directly conjugate-matched to the PA output impedance.

The PA output impedance of IR-UWB transmitters was measured to be 9.5+j22.3Ω at the center frequency of 4.25 GHz. The antenna is therefore designed to present an input impedance of 9.5−j22.3Ω, achieving direct conjugate matching without the need for intermediate matching components. This approach significantly reduces the overall system footprint and eliminates the insertion loss associated with on-chip matching networks.

### 3.2. Design and Implementation of the 8 mm × 10 mm Fractal Antenna

Fractal antennas employ self-similar geometries to achieve wideband operation within a compact form factor. Compared with conventional rectangular patch antennas, which typically exhibit a single resonance mode, fractal structures provide several key advantages for implantable applications [22,23].

#### 3.2.1. Increased Effective Electrical Length

Through iterative geometric patterns at multiple scales, fractal antennas effectively extend the total metal trace length within a confined area. Since the resonant frequency is inversely proportional to the electrical length, fractal antennas can resonate at lower frequencies compared to simple patches of the same physical size.

#### 3.2.2. Inherent Multi-Band/Wideband Characteristics

The self-similarity of fractal geometries, where local and global structures maintain geometric correspondence, naturally supports multi-band or ultra-wideband operation. Each scale of the self-similar sub-structure contributes to a distinct resonance frequency.

#### 3.2.3. Compact Form Factor

The fractal geometry achieves the required bandwidth and frequency coverage within dimensions of 8 mm × 10 mm, meeting the stringent size constraints of implantable neural devices.

**Figure 5:**
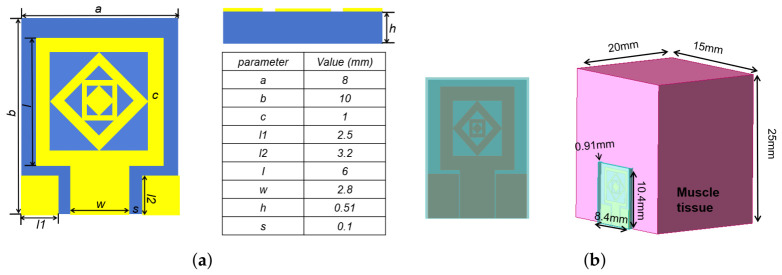
(**a**) Structure of the implantable fractal antenna unit with CPW feeding. (**b**) Silicone-encapsulated antenna implanted in simulated muscle tissue.

The implantable antenna unit, illustrated in Figure 5a, consists of a dielectric substrate (RO4350), a metal ground plane, and a fractal-structured patch. The ground plane and fractal structure are coplanar, fabricated from copper. The fractal structure comprises four iterations: the central diamond serves as the first-order structure, which is scaled down and rotated clockwise to form the second-order structure through nesting, and this process is repeated to generate the third and fourth orders.

A CPW feeding mechanism is adopted due to its advantages in implantable scenarios. The coplanar structure greatly simplifies the manufacturing process. Impedance matching can be flexibly achieved by adjusting the conductor width and gap dimensions. The compact CPW structure is readily combinable with biocompatible materials for in-body implantation.

To ensure proper operation within biological tissue, the antenna is encapsulated in a silicone film (thickness 0.91 mm) and simulated within an HFSS model of human muscle tissue at an implantation depth of 18 mm, as shown in Figure 5b.

**Figure 6:**
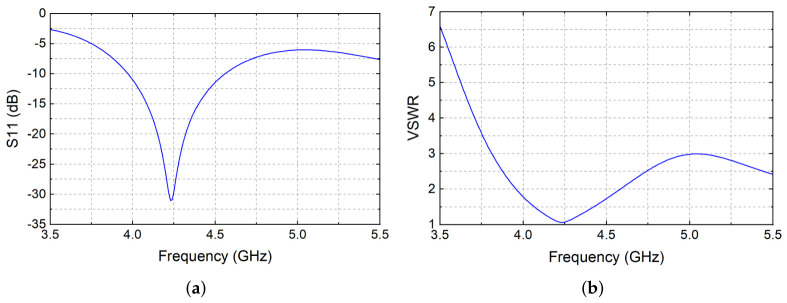
(**a**) Simulated reflection coefficient (S11) of the fractal antenna implanted in muscle tissue. (**b**) Simulated VSWR of the fractal antenna.

The simulated reflection coefficient (S11) and voltage standing wave ratio (VSWR) of the fractal antenna implanted in muscle tissue are shown in Figure 6. The S11 confirms a center frequency of 4.25 GHz with a −10 dB impedance bandwidth of 3.97–4.57 GHz (relative bandwidth of 14%), corresponding to a 600 MHz bandwidth that covers the required UWB operating range. The VSWR remains below 2 across the operating bandwidth, consistent with the reflection coefficient results.

**Figure 7:**
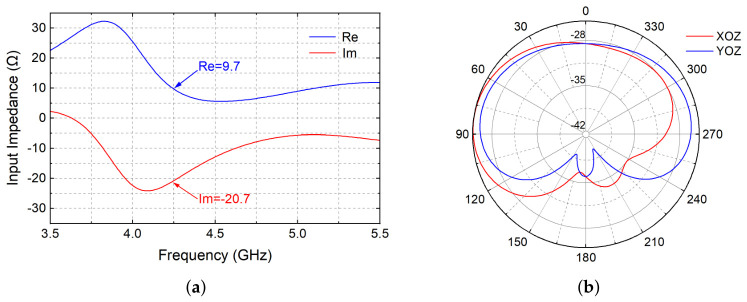
(**a**) Simulated input impedance of the fractal antenna as a function of frequency. (**b**) Simulated gain pattern of the fractal antenna in XOZ and YOZ planes.

The simulated input impedance and gain pattern of the fractal antenna are shown in Figure 7. The input impedance at 4.25 GHz is 9.7−j20.7Ω, closely matching the target conjugate impedance of the PA output. The radiation pattern shows good omnidirectional characteristics in both XOZ and YOZ planes, with a maximum gain of −25 dB in the XOZ plane, which is typical for deeply implanted antennas operating within lossy biological tissue.

The antenna impedance can be tuned through two key geometric parameters of the CPW feed. Increasing the CPW conductor width *w* leads to a gradual increase in both radiation resistance and reactance, with the reactance trending inductive, which can improve the wireless link bandwidth. Increasing the CPW gap *s* while keeping *w* constant results in decreased radiation resistance and reactance, enabling fine-tuned impedance matching toward the PA’s conjugate target.

## 4. Results

### 4.1. Simulation Results of PLL

**Figure 8:**
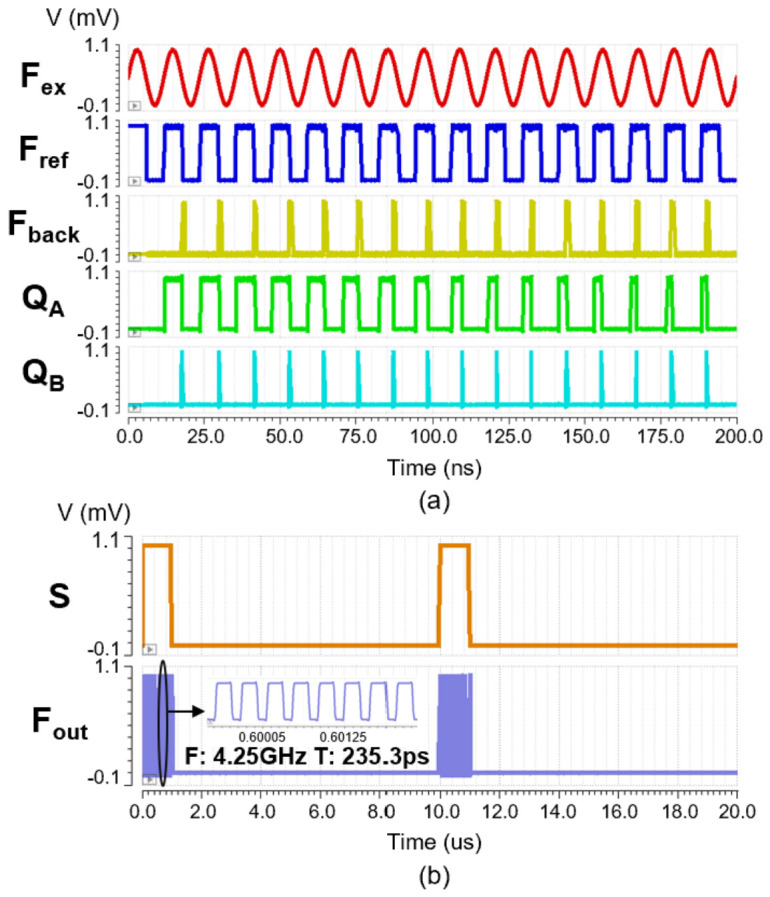
Waveforms of (**a**) PFD sensing the input phase difference and (**b**) PLL output controlled by signal *S*.

The proposed duty-cycled PLL is designed and implemented in TSMC 40 nm CMOS process technology, with a supply voltage of 1.0 V. The carrier frequency of 4.25 GHz is selected due to its lower tissue attenuation for the IR-UWB transmitter. The total division ratio is set to 50, and the reference clock $F_{ref}$ operates at 85 MHz. The control signal *S* is configured with a period of 10 μs, where the calibration phase $T_{1}$ (S=1) lasts for 1 μs, corresponding to a 10% duty cycle. Figure 8 illustrates the frequency difference detected by the PFD between the reference clock and the feedback clock, along with the corresponding PLL output waveform.

**Figure 9:**
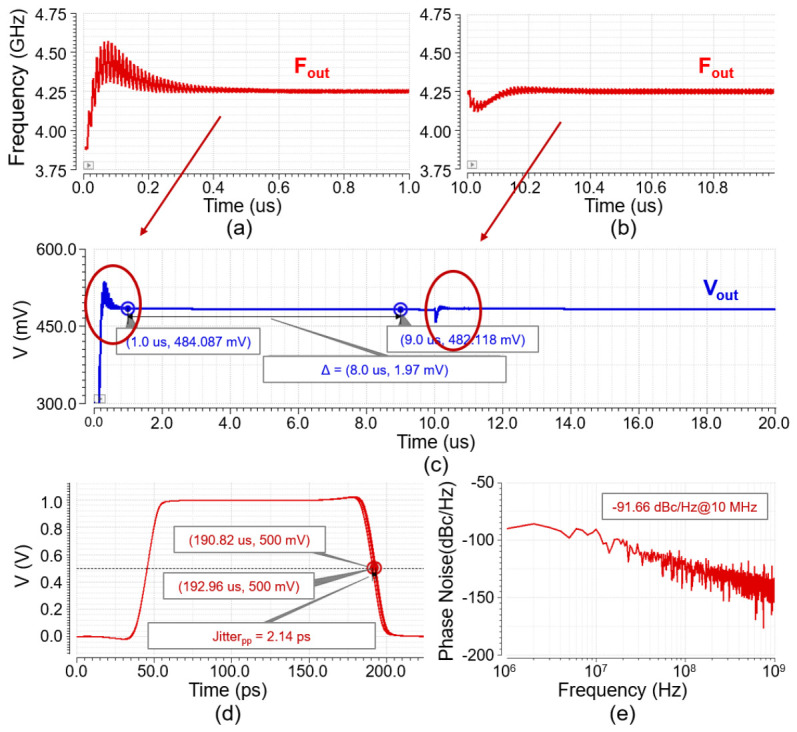
Post-layout simulation results: (**a**,**b**) frequency curve of PLL output signal, (**c**) control voltage $V_{out}$ variation curve of the PLL, (**d**) eye diagram of the PLL output signal, (**e**) phase noise of PLL.

As shown in Figure 9, the system loop enters the locked state within approximately 0.5 μs of startup. The control voltage $V_{out}$ settles at 484 mV upon locking. During an 8 μs silent period, the voltage drop due to leakage is less than 2 mV, corresponding to a carrier frequency deviation of less than 0.5%. This deviation is considered negligible within the wide frequency band of the IR-UWB transmitter.

The PLL achieves a locking frequency of 4.25 GHz with a peak-to-peak jitter (${\mathrm{Jitter}}_{pp}$) of 2.14 ps, characterized over 2100 clock cycles. Notably, the voltage-holding mechanism enables faster relocking in subsequent calibration cycles, reducing the relocking time to approximately 0.2 μs.

### 4.2. Duty-Cycled Power Breakdown and PLL Comparison

**Figure 10:**
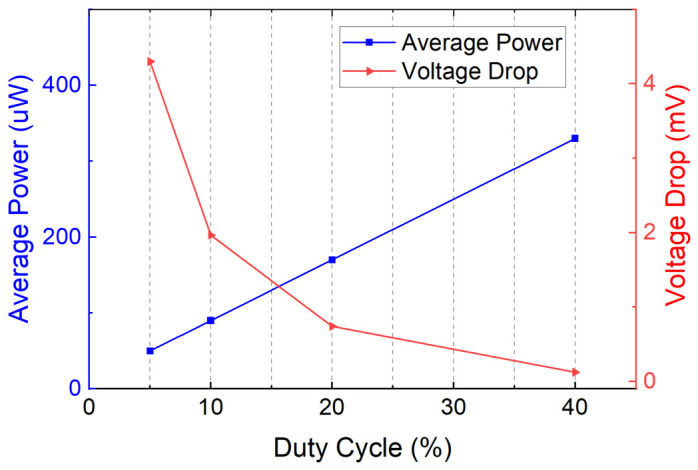
Average power and voltage drop with different duty cycles.

Figure 10 presents the average power and voltage drop with different duty cycles. The average power increases proportionally with duty cycle since the loop remains active solely during S=1. At low duty cycles, the voltage drop becomes substantial and the carrier frequency may drift beyond acceptable limits for narrowband systems. The 10% duty cycle represents an optimal balance, offering significantly reduced drop compared to 5% while maintaining sub-hundred microwatt power consumption. Further increasing to 20% or 40% diminishes drop at the cost of elevated power budget. For IR-UWB transmitters, where the passband is inherently wide, minor residual drift remains tolerable, making 10% a practical operating point.

**Figure 11:**
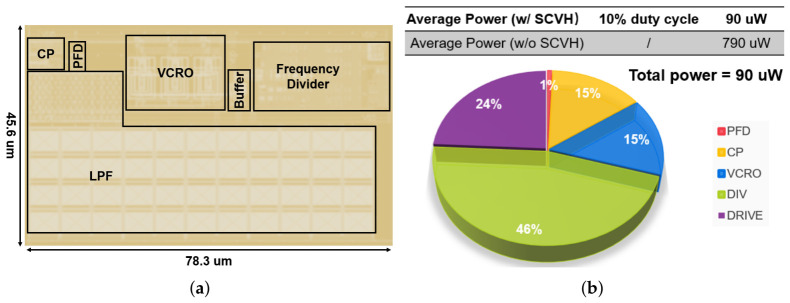
(**a**) Layout of the proposed PLL. (**b**) PLL power breakdown.

Figure 11a presents the layout of the proposed PLL. The low-pass filter occupies the largest area due to the integrated capacitance required for loop filtering, with the core area measuring only 45.6 × 78.3 μm^2^ (0.0035 mm^2^). As depicted in Figure 11b, with SCVH enabled at 10% duty cycle, the average power consumption reaches 90 μW. In contrast, the same loop without SCVH draws approximately 790 μW in simulation, representing a ninefold reduction or 89% savings. The power breakdown reveals that the frequency divider consumes the largest portion, approaching half of the total consumption, followed by the buffer or drive stage. The ring oscillator and charge pump exhibit comparable contributions, indicating that timing generation and the feedback path dominate the power budget rather than the phase detector itself.

Table 1 summarizes the performance of the implemented PLL and provides a comparison with other designs. A figure of merit (FoM) is defined as the ratio of power consumption to operating frequency (mW/GHz), which allows for a fair comparison of architectural efficiency independent of the target frequency. The proposed PLL demonstrates a significant reduction in both power consumption and silicon area, achieving a superior FoM compared to its counterparts.

### 4.3. In Vitro Wireless Measurement Through Biological Tissue

**Figure 12:**
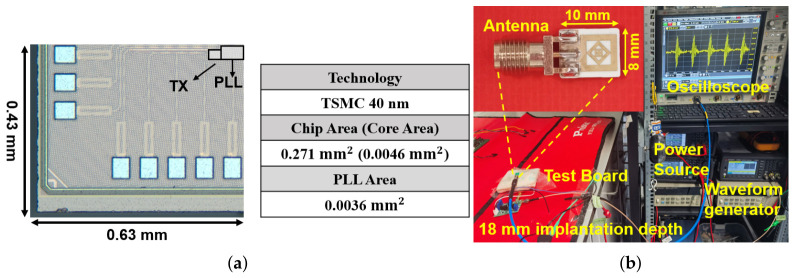
(**a**) Die micrograph of the proposed IR-UWB TX with integrated duty-cycled PLL, fabricated in TSMC 40 nm CMOS. (**b**) Measurement setup for in vitro wireless testing.

To validate the complete transmitter system integrating the duty-cycled PLL with the co-designed fractal antenna, in vitro wireless experiments were conducted on the same TSMC 40 nm CMOS chip, with a supply voltage of 1.0 V. As illustrated in Figure 12a, the total chip area is 0.63×0.43 mm^2^ (0.271 mm^2^), while the transmitter core occupies 0.0046 mm^2^ and the PLL core occupies 0.0036 mm^2^.

As shown in Figure 12b, the co-designed fractal antenna, measuring 8 mm × 10 mm, is connected to the TX output through an SMA connector. The KEYSIGHT 33600A Arbitrary Waveform Generator (Santa Rosa, CA, USA) provides a 100 MHz rectangular pulse train as the input data stream to the TX. The test board is placed inside fresh porcine tissue at an implantation depth of 18 mm that includes both skin and subcutaneous fat layers. A real-time oscilloscope captures the received pulses on the external side.

**Figure 13:**
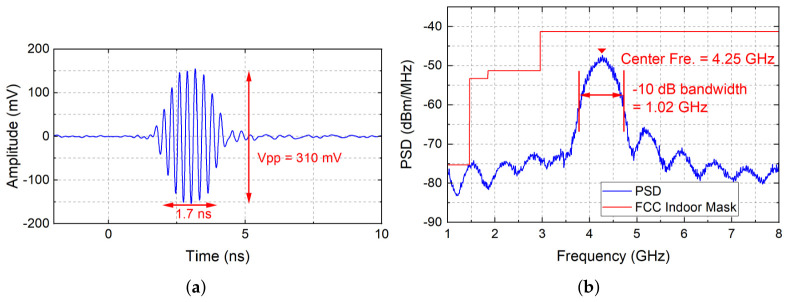
(**a**) Measured output pulse waveform of the IR-UWB TX with PLL-generated control voltage. (**b**) Measured PSD of the TX output signal with the integrated duty-cycled PLL. Center frequency = 4.25 GHz, −10 dB bandwidth = 1.02 GHz.

Figure 13a shows the peak-to-peak amplitude of TX can reache 310 mV and the pulse duration is 1.7 ns, when the PLL supplies the control voltage to the VCRO_TX. As depicted in Figure 13b, the measured power spectral density (PSD) of the TX output signal shows a center frequency located at 4.25 GHz, matching the target operating frequency of the PLL. The −10 dB bandwidth measures 1.02 GHz, covering the 3.1–6 GHz UWB band allocated by the FCC. The entire spectral envelope remains below the FCC indoor mask of −41.3 dBm/MHz, confirming regulatory compliance. This result directly validates the frequency calibration capability of the proposed duty-cycled PLL.

**Figure 14:**
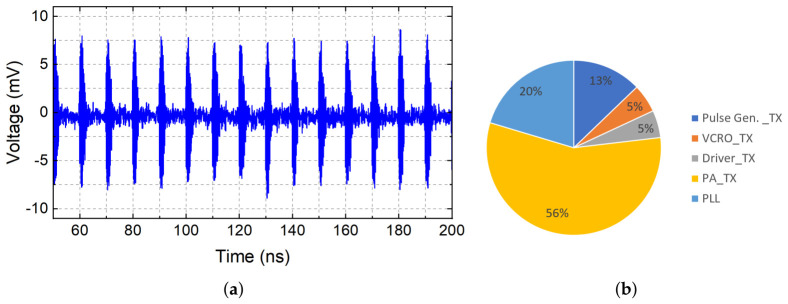
(**a**) Received time-domain waveform at 100 Mbps OOK through 18 mm porcine tissue using the co-designed fractal antenna. (**b**) Power breakdown of the complete IR-UWB TX system including the duty-cycled PLL.

Figure 14a presents the received time-domain waveform captured at the external antenna after the signal has traveled through 18 mm of porcine tissue. The received pulses are clearly distinguishable from the noise floor. Adjacent pulses show consistent amplitude and spacing across the full observation window of about 150 ns, confirming stable timing from the PLL-locked carrier.

Figure 14b shows the power breakdown of the TX including the proposed duty-cycled PLL. Compared with the standalone TX breakdown, the PLL adds roughly one sixth to the total system power. This overhead is modest given that it replaces the need for an external precision voltage source and provides autonomous frequency calibration through the SCVH mechanism. At a 10% PLL duty cycle, the effective contribution of the PLL to the average TX power drops further, as the PLL is only active during calibration intervals and draws near-zero current during the silent phase.

Table 2 summarizes the measured performance and provides a comparison with state-of-the-art IR-UWB TX designs. A Figure-of-Merit (FoM) is defined as the ratio of normalized energy efficiency to peak-to-peak amplitude, where a lower FoM indicates better overall performance. The proposed TX consumes only 0.58 mW including the duty-cycled PLL, which is the second lowest among all compared designs. Only ref. [26] achieves a lower TX power of 0.42 mW, but that design relies on an external clock and without on-chip frequency calibration. In contrast, this work integrates a complete duty-cycled PLL that consumes only 90 μW, 76× lower than the 6.9 mW conventional CPPLL reported in [27]. The co-designed fractal antenna further eliminates the area-intensive on-chip matching network required by conventional 50 Ω designs. Overall, the proposed design achieves a FoM of 9.3 pJ/(b·V), comparable to the best values in the table, while being the only design that simultaneously offers low-power on-chip frequency calibration, through-tissue transmission at 18 mm, and constant energy efficiency across a wide data rate range.

## 5. Conclusions

This paper presented a low-power, duty-cycled phase-locked loop (PLL) and a co-designed compact fractal antenna architecture tailored for neural implants. By integrating a switch-controlled voltage-holding (SCVH) module, the proposed design enables periodic calibration while maintaining voltage stability during silent phases, achieving very low power consumption while maintaining a stable carrier frequency.

The PLL employs a dual-oscillator scheme in which one VCRO operates within the feedback loop while a second, identical VCRO generates the transmitter carrier. Post-layout simulation in TSMC 40 nm CMOS confirms a locking frequency of 4.25 GHz, a locking time of 0.5 μs, a peak-to-peak jitter of 2.14 ps, and an average power consumption of 90 μW. This represents an 89% power reduction compared to a continuously active PLL and yields a figure of merit of 0.021 mW/GHz, which is more than 10× better than prior PLL designs operating at comparable frequencies.

A compact fractal antenna (8 mm × 10 mm) was co-designed with a CPW-fed structure to directly present the conjugate impedance of the PA output (9.7−j20.7Ω at 4.25 GHz). This co-design approach eliminates the on-chip matching network entirely, reducing the total system footprint. The antenna achieves a −10 dB impedance bandwidth of 600 MHz (3.97–4.57 GHz) and a VSWR below 2 across the operating band when implanted in simulated muscle tissue.

The integrated TX-PLL system was fabricated and measured through in vitro porcine tissue experiments. The measured output pulse maintains 310 mV peak-to-peak amplitude and 1.7 ns pulse width. The measured PSD confirms a center frequency of 4.25 GHz with a −10 dB bandwidth of 1.02 GHz, validating the frequency calibration accuracy of the duty-cycled PLL. The complete TX system including the PLL consumes 0.58 mW, and the comparison with state-of-the-art IR-UWB transmitters shows competitive energy efficiency (2.9 pJ/b across 10–200 Mbps), the lowest PLL power among designs with on-chip frequency calibration, and a favorable composite figure of merit of 9.3 pJ/(b·V).

## Figures and Tables

**Table 1:** PLL performance comparison.

Parameter	This Work ^*b*^	[24] ^*b*^	[16]	[25]
Process (nm)	40	90	65	65
Supply Voltage (V)	1.0	0.6	1.2	N/A
Frequency (GHz)	4.25	0.915	4.5	2.0
Silent Mode	Yes	Yes	No	No
${\mathrm{Jitter}}_{pp}$ (ps)	2.14	3.39	N/A	8.03
Core Area (mm^2^)	0.0035	0.109	0.16 ^*c*^	0.049
Power (mW)	0.09 ^*d*^	0.2	3.78	9.0
FOM ^*a*^ (mW/GHz)	0.021	0.218	0.84	4.5

^*a*^ FOM = Power/Frequency (mW/GHz). ^*b*^ Post-layout simulation. ^*c*^ Estimated from chip micrograph. ^*d*^ 10% duty-cycle.

**Table 2:** Performance comparison with state-of-the-art IR-UWB TXs.

Parameter	This Work	[26]	[27]	[28]	[8]	[29]	[30]
Process (nm)	40	65	65	40	28	65	180
Modulation	OOK	OOK	OOK	D16PPM+PWM +DBPSK	4PPM+8PSK +4PAM	D-MPPM	OOK
Bandwidth (GHz)	3.1–6	5–8	3.1–6	3.1–5	6–9	3.1–5	3.1–6
Max. Data Rate (Mbps)	200	400	800	1800	1660	500	200
Amplitude, $V_{pp}$ (mV)	310	150	400	260	230	400	260
TX Power (mW)	0.58	0.42 ^*d*^	13.2	4.09	9.69	7.0	4.0
Energy Eff. (pJ/b)	2.9@10–200 Mbps	1.05	16.5	2.3	5.8	14	20
Tissue Type	18 mm skin/fat	N/A	15 mm skin/fat	18 mm skin/fat	15 mm skin/fat	No Tissue	N/A
Frequency Calibration	Duty-cycle CPPLL	N/A	Conventional CPPLL	N/A	N/A	N/A	N/A
PLL Phase Noise (dBc/Hz)	−91.66@10 MHz ^*e*^	N/A	−96.77@10 MHz	N/A	N/A	N/A	N/A
PLL Power (mW)	0.09	N/A	6.9	N/A	N/A	N/A	N/A
Area (mm^2^)	0.271, 0.0046 ^*c*^	0.12 ^*c*^	2.0	0.058 ^*c*^	0.155 ^*c*^	38 ^*b*^	0.021 ^*c*^
FoM ^*a*^	9.3	7.0	41.25	8.85	25.22	35.0	76.92

^*a*^ FoM = Normalized Energy Efficiency/$V_{pp}$ [pJ/(b·V)]. ^*b*^ Transceiver. ^*c*^ Core area only. ^*d*^ External clock. ^*e*^ Simulation.

## Data Availability

Data are contained within the article.
